# Annotations

**Published:** 1886-10-30

**Authors:** 


### ANNOTATIONS.

**The Out-Patient Question.** ANYONE who has endeavoured to ascertain from the hospitals the cost of an out-patient, must have been struck by the very diverse opinions which prevail on this subject. One London hospital, with 50,000 out-patients, returns the cost at 2*s*. 9*d*. per head; another, with 24,000 out-patients, puts it at 2*s*. per head; whilst another, with the same number of out-patients, makes it only 7*d*. per head. In the provinces, one of the largest infirmaries, having 15,000 out-patients, states the cost of each to be 6*s*. 10*d*.; while another county hospital, with 1,700 out-patients, returns it at 10*s*. 11*d*. Those who have given impartial consideration to the subject, however, believe that an average cost of 1*s*. per head, per out-patient, rather exceeds, than otherwise, the actual cost incurred by the general hospitals in these cases. In any case, there was much reason in the contention on the part of the working men of Burton last week that the out-patients did not cost the infirmary £1 15*s*. each, the sum paid by the Saturday Committee for each out-ticket they obtained from that institution. Mr. Cross, one of the Committee, gave instances where they had had to give out-patients' tickets to men employed at his works, where the relief afforded could not be valued at probably more than 1*s*. It appears to be the practice at Burton to insist upon the production of an out-patients' ticket for each case of emergency or accident, however slight. This is contrary to the practice of the hospitals, as a rule, all over the country; and although the Saturday Committee has never had to suffer, because the Hospital Committee have always shown commendable liberality, we are of opinion that it is desirable, in the interests of the Burton Infirmary, that they should alter their rule so as to admit of the treatment of accident and emergency cases free, without ticket. This would confer great benefits on the poor, increased popularity on the infirmary, and, consequently, considerably increase voluntary contributions.

**The Exhibition of Competition Plans.** The Committee of the new infectious hospital at Liverpool have caused some dissatisfaction by refusing a petition from the Liverpool architects competing, to permit the exhibition of all the plans sent in for competition. Of course, it would be improper to allow any competition drawings to be publicly exhibited until the assessor has given his award. When this has once been done, it is not only fair, but highly desirable on public grounds, that the wholeof the plans sent in, in such cases, shall be publicly exhibited. In a recent competition for the new lunatic asylum at Derby, the plans were exhibited by the Corporation, and eminent experts came from long distances in order to see how much novelty and improvement might fairly be credited to those competing. In all public institutions the best plan should be accepted, and such a result is sure to be contributed to by allowing the public, after the award, to see and judge for themselves. Such competitions also afford an occasion which should not be neglected, to widen the interest taken in hospitals, their construction and work. On these grounds, and on others which might be mentioned, we hope, as the Liverpool plans have not yet been returned, that the Council or the Committee will reconsider their decision, and comply with the reasonable request of the Liverpool architects.

**Incurable and Chronic v. Acute Disease.** Certain remarks made by Dr. Goyder at the opening of the bazaar in aid of the Bradford Children's Hospital, last week, have been misunderstood. It appears that the Bradford Infirmary contains an excellent, large, and well-arranged children's ward, with ample accommodation for the treatment of acute cases of disease and injury. The new Children's Hospital at Bradford, on the other hand, is intended to be a home for incurable cases, and for this reason its establishment is most desirable. In these circumstances, Dr. Goyder expressed a hope that a right direction would be secured for both the Children's Hospital and the Bradford Infirmary, which was wrongly taken to mean that he was unable to sympathise with two equally deserving institutions. The objectors failed to realise the difference between acute and chronic or incurable diseases, and the necessity for treating each in separate hospitals. An ordinary hospital, which admits patients suffering from acute diseases, finds it necessary to have a rule limiting the number of days during which each case shall be resident within its wards. The function of a hospital, rightly understood, is to cure the greatest number of patients, with the fewest number of beds, in the shortest possible time. Hence an ordinary hospital is no place for chronic or incurable cases, because such patients have necessarily to be provided for for a lengthened period, if not for the rest of their natural lives. There can be no antagonism, therefore, between the children's ward in the acute hospital, and the institution for incurable children, at Bradford, and Dr. Goyder's remarks were not only reasonable but apposite. The discussion shows the importance of providing accommodation, in separate buildings, for a certain class of incurable and chronic cases, which at present fare badly, from the want of a sufficient number of such institutions.

